# Supplementary material for: Tumor Regression upon Intratumoral and Subcutaneous Dosing of the STING Agonist ALG-031048 in Mouse Efficacy Models
Source: Int J Mol Sci. 2023 Nov 13;24(22):16274. doi: 10.3390/ijms242216274 (PMC10671074; doi:10.3390/ijms242216274)
Supplement: Supplementary file 1 [file ijms-24-16274-s001.zip › ijms-2693711-supplementary.pdf]

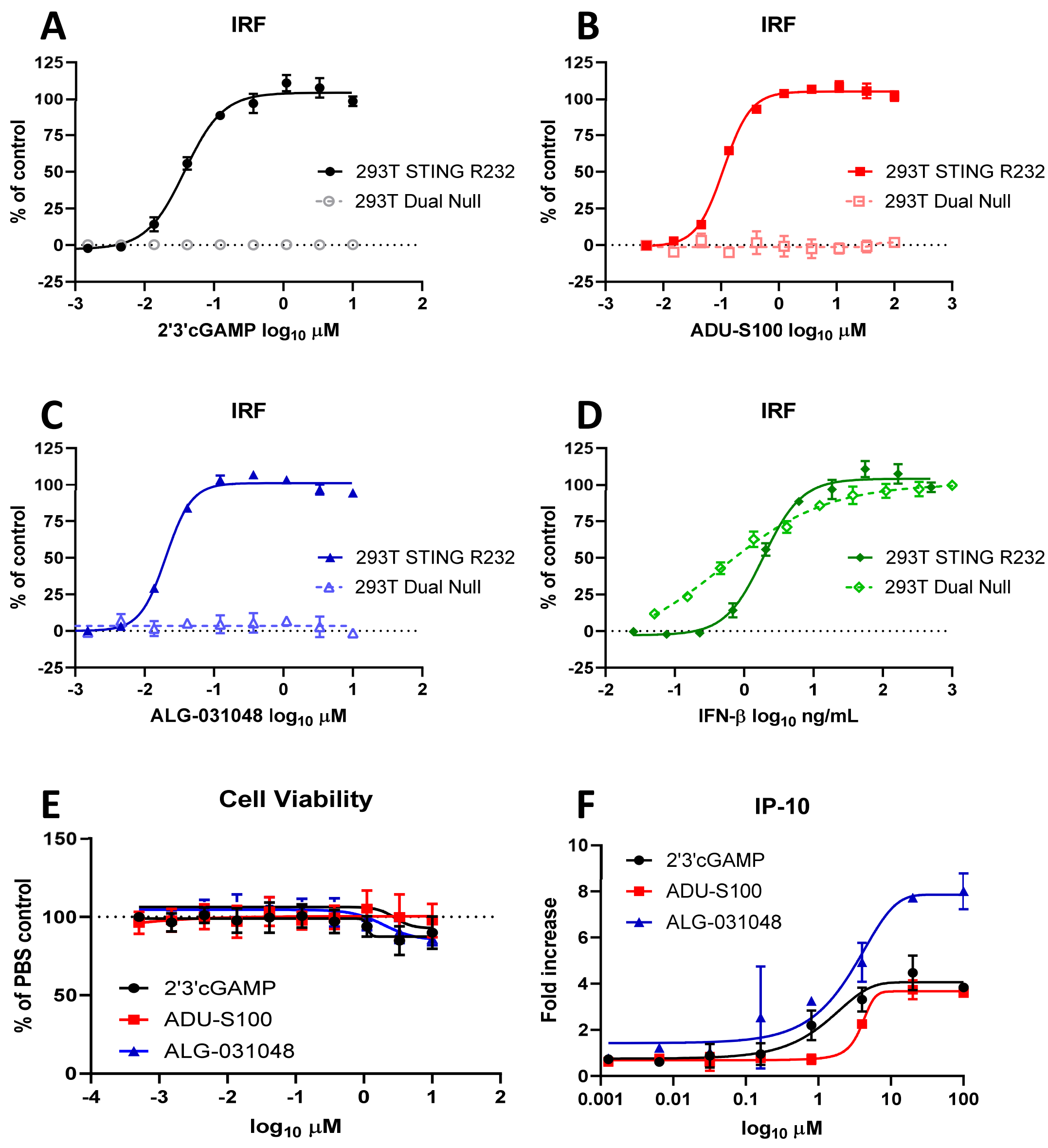

Figure S1. Cellular activation induced by 2′3′ cGAMP (A), ADU-S100 (B) and ALG-031048 (C) but not IFN-β (D) is dependent on the expression of STING. HEK 293 reporter cells expressing the STING variant R232 (solid line) or control cells lacking STING expression (dashed line) were treated with increasing concentrations of the STING agonists or IFN-β. After 24 hours, IRF reporter activity was assessed. Shown are the mean of at least three biological replicates and standard deviation as error bars. E: Cell viability of HEK 293 reporter cells treated with STING agonists at concentrations up to 1000 nM. F: ALG-031048 induced release of IP-10 in THP-1 cells.

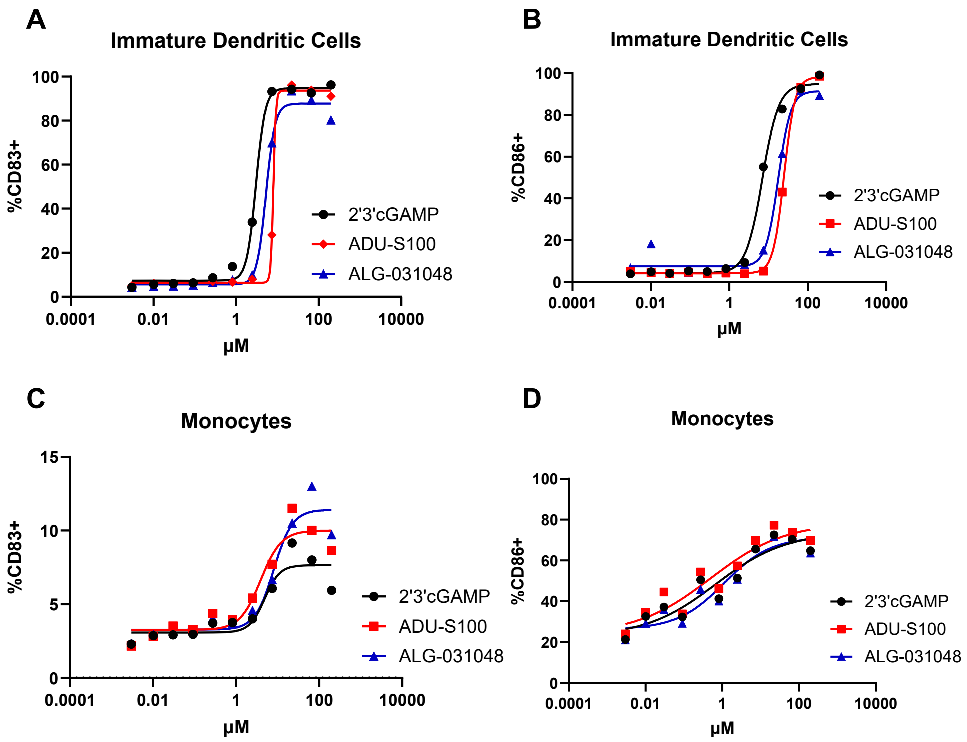

Figure S2. Dose-dependent upregulation of CD83 (A, C) and CD86 (B, D) on immature dendritic cells 72h (A, B) and monocytes 24h (C, D) after stimulation with ALG-031048 (blue), 2'3' cGAMP (black) and ADU-S100 (red).

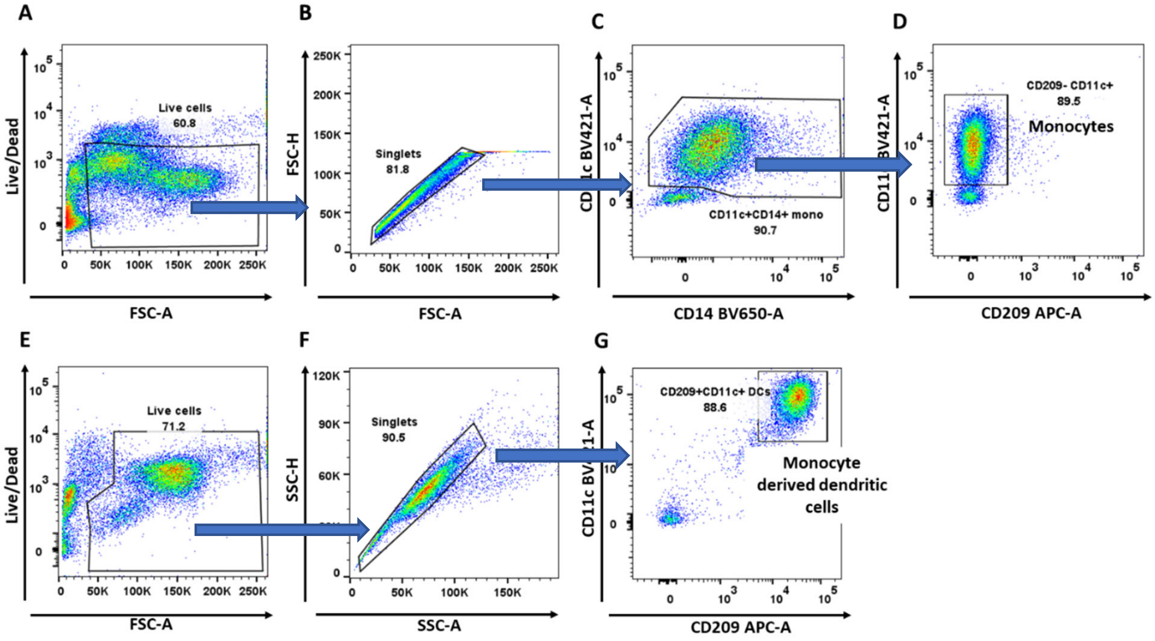

Figure S3. Gating strategy for flow cytometry analysis used in Figure 3 and Figure S2. A, B, C and D: CD14+ monocytes were enriched using negative selection. Gating strategy to identify CD14$^+$ CD209$^-$ CD11c$^+$ monocytes A: Live cells were selected based on live/dead staining and cell size. B: Dot plot of size FSC-A versus FSC-H for doublet exclusion, selecting singlet cells. C: Selecting CD11c$^+$ cells in dot plot of CD11c versus CD14. D: Dot plot of CD209 versus CD11c, selecting CD14$^+$ CD209$^-$ CD11c$^+$ monocytes and excluding antigen-presenting cells. E, F and G: Enriched monocytes were differentiated using human IL-4 and human GM-CSF. Gating strategy to identify CD209$^+$

CD11c⁺ monocyte-derived dendritic cells. E: Live cells were selected based on live/dead staining and cell size. F: Dot plot of size SSC-A versus SSC-H for doublet exclusion, gating on singlet cells. G: Dot plot of CD209 vs CD11c to select CD209⁺CD11⁺ high monocyte-derived dendritic cells.

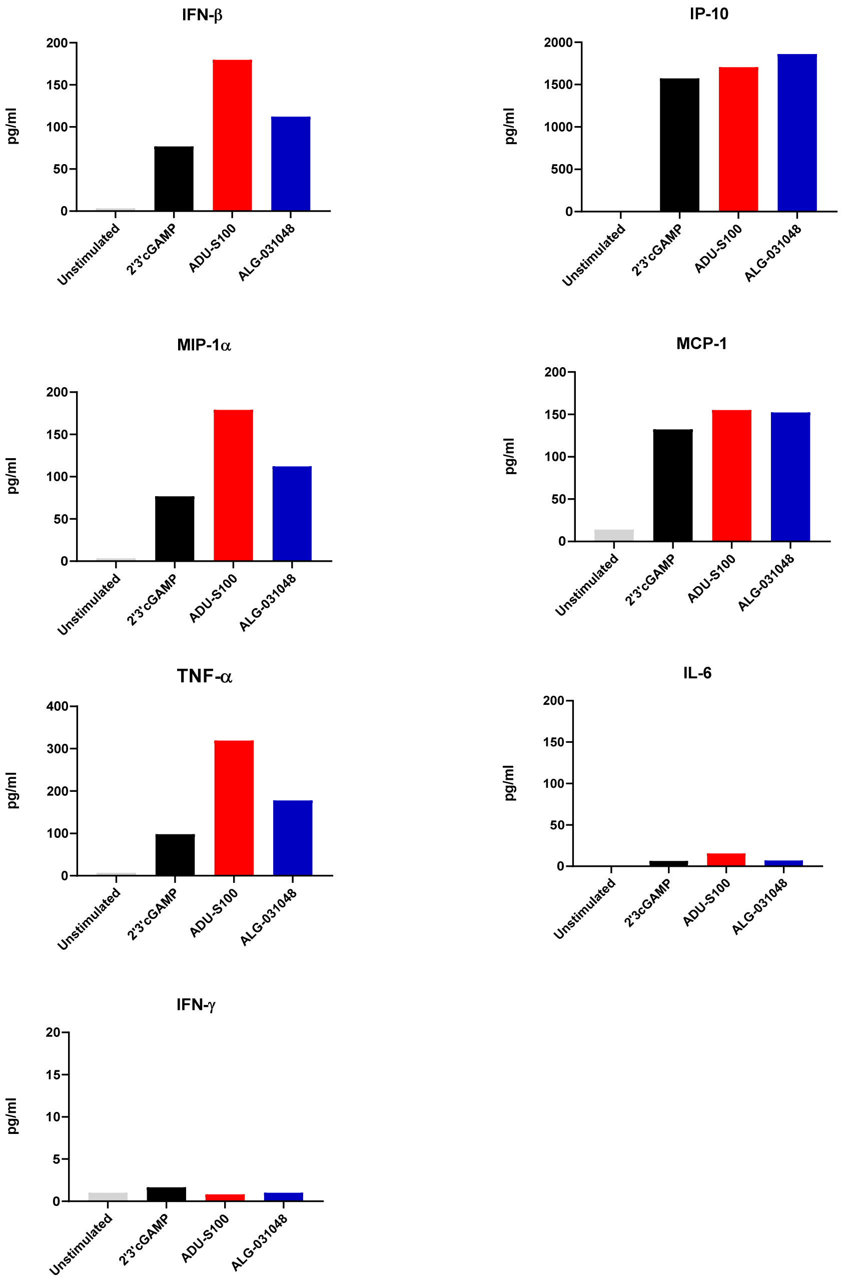

Figure S4. Increased release of cytokines from iDCs after stimulation for 24 hours with 50 μM of the STING agonists ALG-031048, 2′3′ cGAMP, and ADU-S100.

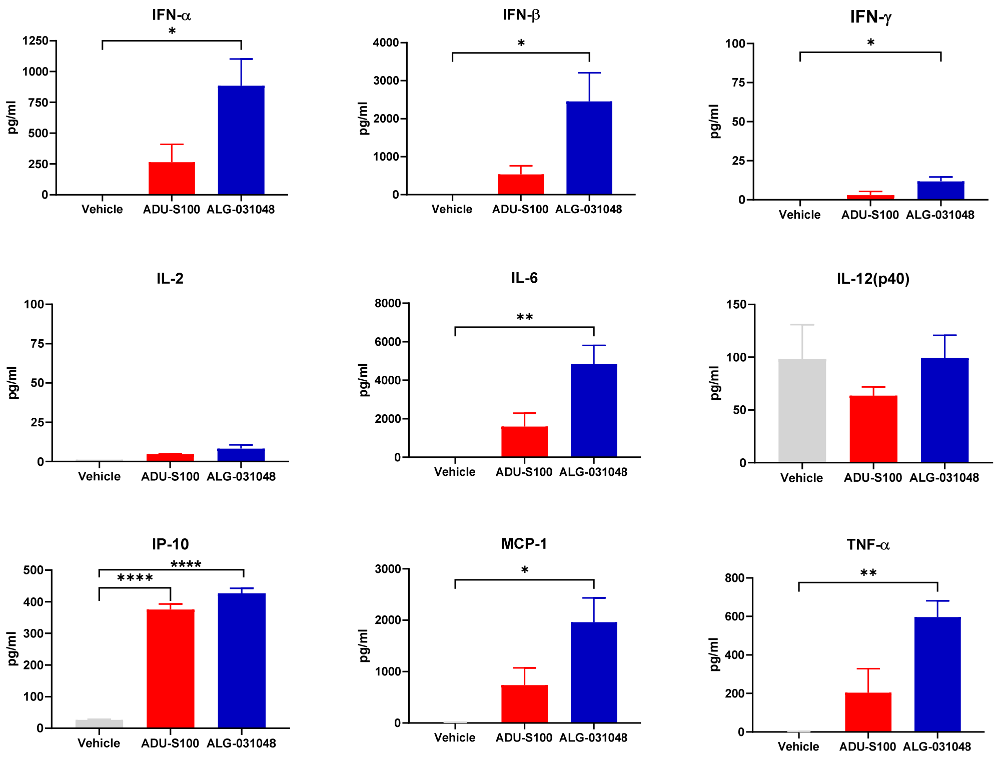

Figure S5. Plasma cytokine levels in in female BALB/c mice bearing SC CT26 tumors receiving one IT dose of 100 μg ADU-S100 or ALG-031048, at a mean TV of 116 mm^3. Cytokine concentrations were analyzed 4 hours after dosing. Only statistically significant differences (unpaired, parametric t test) are indicated by brackets.

A

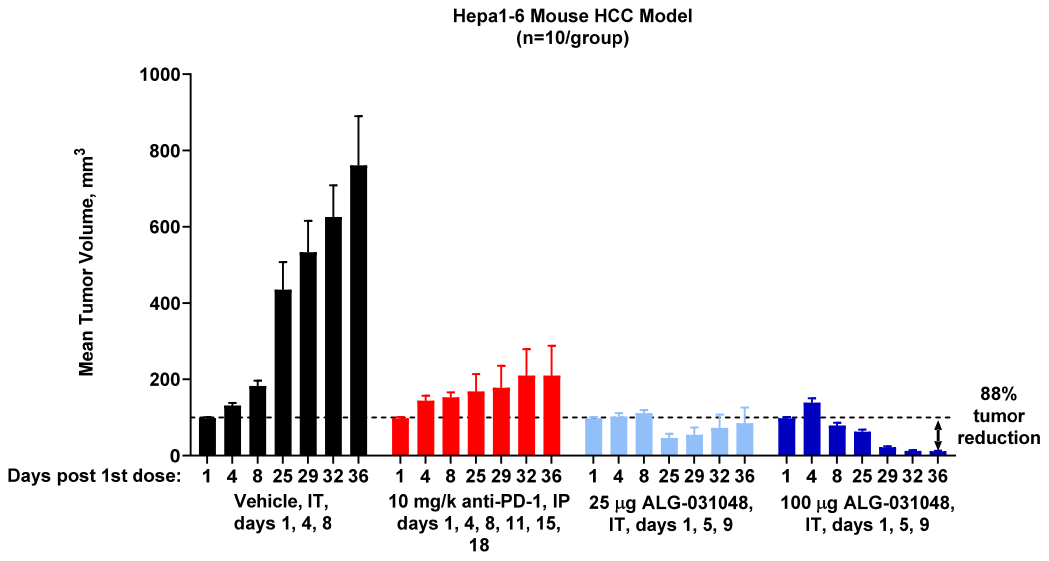
Hepa1-6 Mouse HCC Model
(n=10/group)
Mean Tumor Volume, mm3
1000
800
600
400
200
0
88%
tumor
reduction
Days post 1st dose:
1 4 8 25 29 32 36
Vehicle, IT,
days 1, 4, 8
10 mg/k anti-PD-1, IP
days 1, 4, 8, 11, 15,
18
25 µg ALG-031048,
IT, days 1, 5, 9
100 µg ALG-031048,
IT, days 1, 5, 9

B

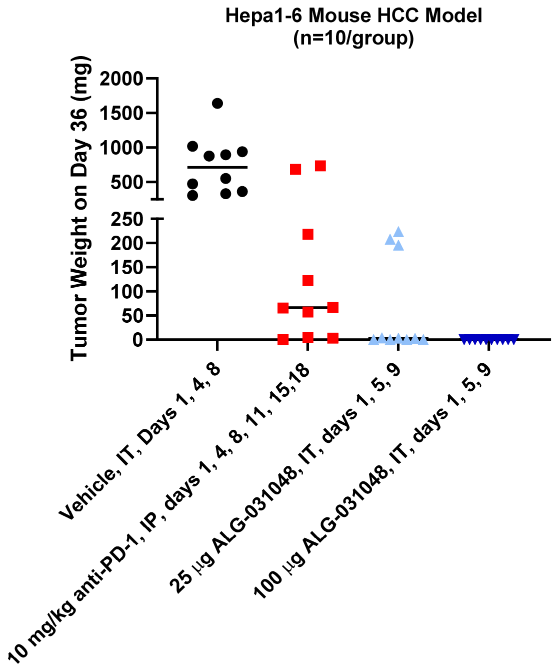
Hepa1-6 Mouse HCC Model
(n=10/group)
Tumor Weight on Day 36 (mg)
2000
1500
1000
500
250
200
150
100
50
0
Vehicle, IT, Days 1, 4, 8
10 mg/kg anti-PD-1, IP, days 1, 4, 8, 11, 15,18
25 µg ALG-031048, IT, days 1, 5, 9
100 µg ALG-031048, IT, days 1, 5, 9

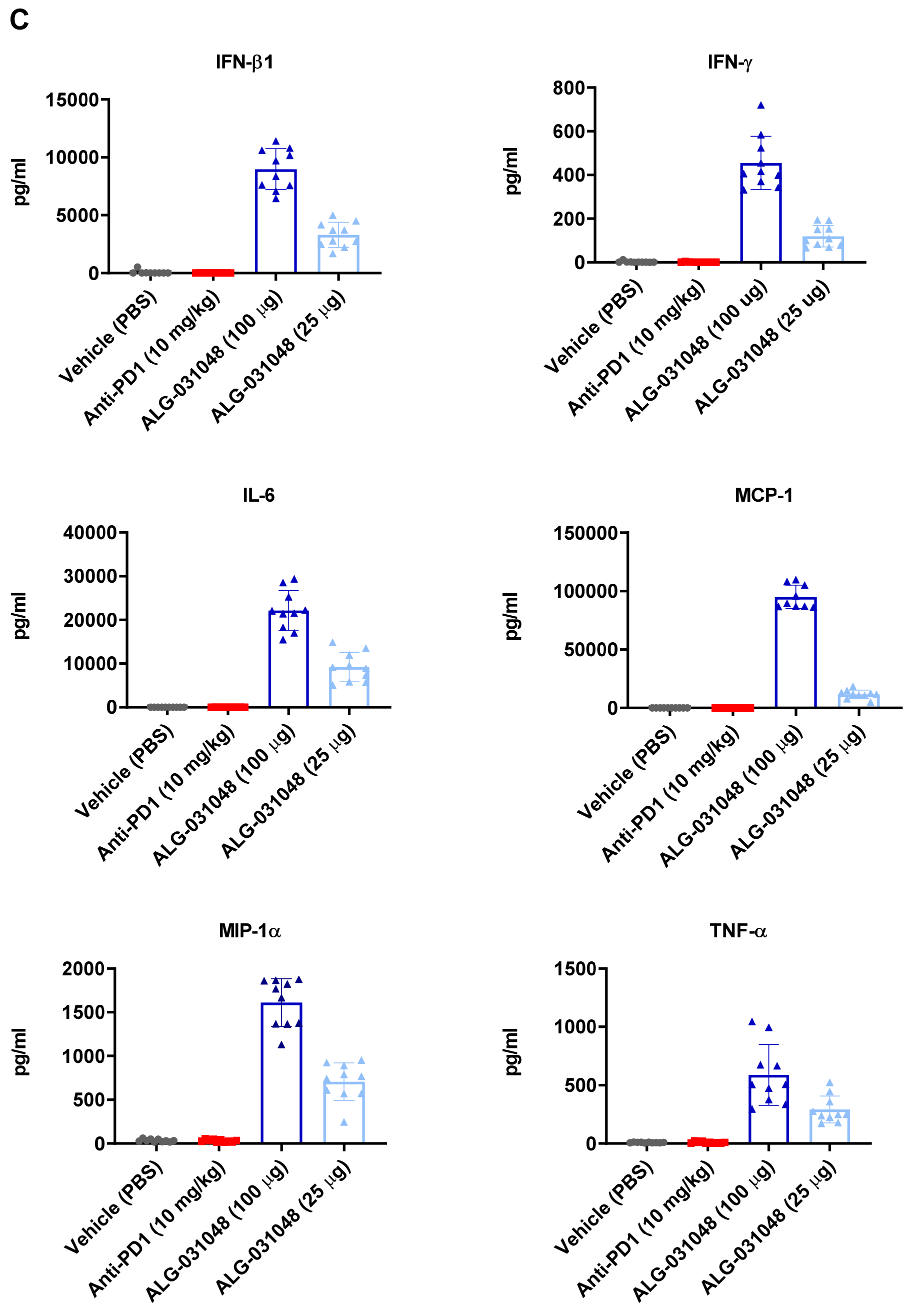

Figure S6. A: Tumor regression in Hepa1-6 tumor-bearing BALB/c mice upon IT treatment with three doses of 25 μg (light blue) or 100 μg ALG-031048 (dark blue), in comparison to treatment with 10 mg/kg anti-PD-1 antibody twice per week, 6 doses total (red). Vehicle-treated animals are shown in black; shown are the mean tumor volumes ± standard deviation for each group of 10 animals. Irregular, crater-shaped tumors prevented accurate tumor volume measurement between Days 8-25. Treatment started when tumors reached an average volume of 98 mm^3. Statistical analysis provided in Table S4. B: Tumor weight for each group at the end of the study. Statistical analysis provided in Table S5. C: Plasma cytokine levels 6 hours after the first dose. Each symbol represents one animal as well as the mean of the group (n=10). Unpaired t-test was used to compare different groups: all groups for all cytokines were statistically significant from each other with P-values of < 0.0001 with the following exceptions: vehicle vs anti-PD-1 were non-significant (all cytokines); the P-value for ALG-031048 (100 μg) vs ALG-031048 (25 μg) was 0.0041 for TNF-α.

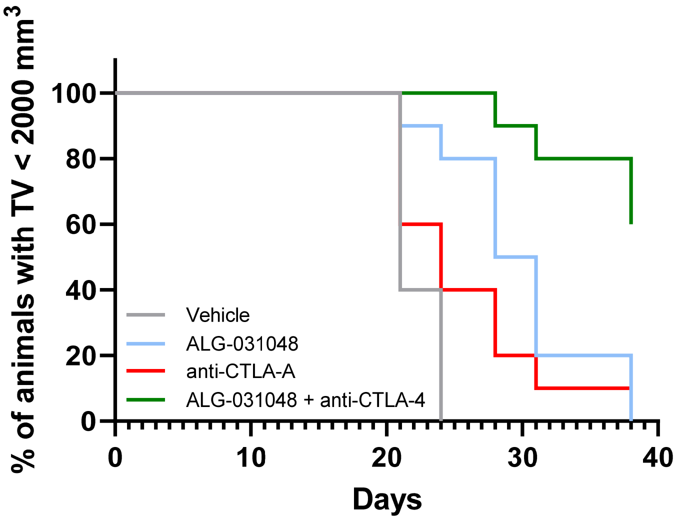

Figure S7. Improved anti-tumor activity of CT26 tumor-bearing mice with the combination treatment of anti-CTLA-4 and ALG-031048. Kaplan-Meier plot showing the percentage of animals with a tumor volume <2000 mm^3. Animals treated with vehicle (grey) or 25 µg ALG-031048 dosed intratumorally on Days 1, 4, and 7 (light blue), or 5 mg/kg anti-CTLA-4 on Day 1 followed by 1 mg/kg anti-CTLA-4 on Days 4 and 7 (red) or a combination of ALG-031048 and anti-CTLA-4 (green). Treatment started at a median tumor volume of 108 mm^3, 13 days post-implantation. Tumor volumes were measured every 3 days.

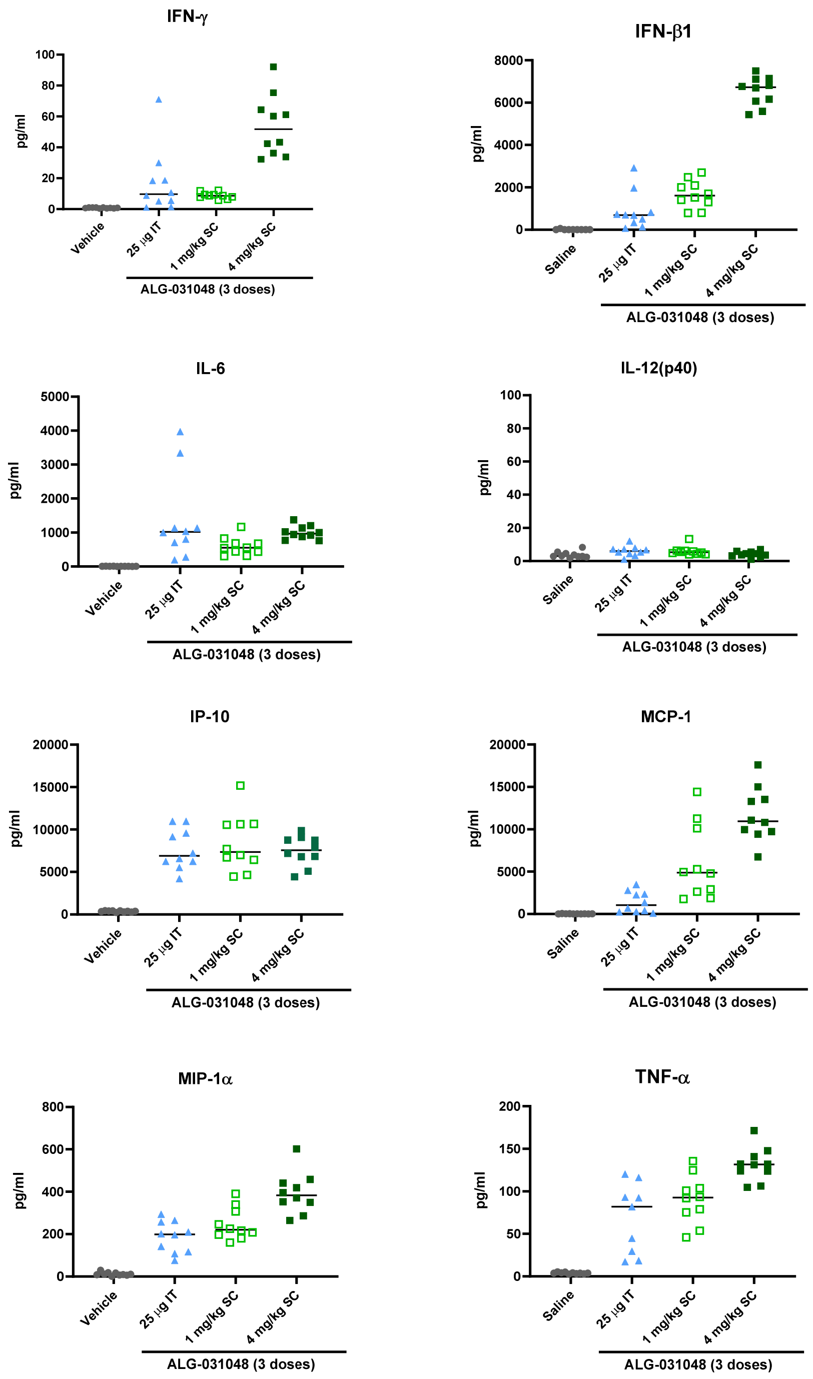

Figure S8. Cytokine induction in CT26 tumor-bearing mice upon SC administration of ALG-031048. 10 mice per group were treated with 3 doses 3 days apart. Plasma samples were collected 6 hours after the last dose. Statistical analysis is provided in Table S9.

Table S1. Binding of 2′3′ cGAMP, ADU-S100 and ALG-031048 STING R232 in a thermal shift assay as well as in the HEK 293 STING R232 and HEK 293 STING null cell line. The data shown are mean±standard deviation.

	**2′3′ cGAMP**	**ADU-S100**	**ALG-031048**	**IFN-β**
**Thermal Shift Assay**				
K_d [μM]	2.76±0.79 (n=20)	4.39±0.50 (n=3)	2.71±0.81 (n=6)	n.d.
Maximum shift °C	16.8±1.66 (n=20)	10.4±0.49 (n=3)	13.8±2.98 (n=6)	n.d.
**Reporter Cell Assay EC_{50} [μM]**				
STING R232 IFN-ß	0.123±0.205 (n=37)	0.337±0.547 (n=39)	0.132±0.186 (n=18)	n.d.
STING R232 IRF	0.030±0.017 (n=36)	0.101±0.065 (n=37)	0.029±0.015 (n=18)	0.84±0.86 (n=3)
STING Dual Null IRF	>10 (n=4)	>10 (n=4)	>100 (n=2)	8.55±9.95 (n=6)

Table S2. Activation of iDCs and monocytes after treatment with various STING agonists.

	**Immature Dendritic Cells**		**Monocytes**	
	**EC_{50} CD86 [μM]**	**EC_{50} CD83 [μM]**	**EC_{50} CD86 [μM]**	**EC_{50} CD83 [μM]**
2′3′ cGAMP	7.0	3.0	0.67	4.9
ADU-S100	25.0	8.0	0.49	3.9
ALG-031048	17.8	5.3	1.12	8.1

Table S3. Statistical analysis (P-value) of CT26 study in support of Figure 4. The time (days) between start of treatment and endpoint (TV ≥2000 mm^3) was used to compare groups. If the endpoint was not reached by the end of the study, the last day of measurement (Day 40) was used instead.

	**P-Value**				
	**Vehicle**	**25 μg ADU-S100**	**100 μg ADU-S100**	**25 μg ALG-031048**	**100 μg ALG-031048**
Vehicle		<0.001	<0.001	<0.001	<0.001
25 μg ADU-S100			0.086	0.398	0.033
100 μg ADU-S100				0.195	>0.999
25 μg ALG-031048					0.087

Table S4. Statistical analysis (P-value) of tumor volumes in Hepa1-6 study presented in Figure S6A. The tumor volume on the last day of measurement (Day 36 after treatment start) was used to compare groups.

	**P-Value**			
	**Vehicle**	**10 mg/kg anti-PD-1**	**25 μg ALG-031048**	**100 μg ALG-031048**
Vehicle		0.002	<0.001	<0.001
10 mg/kg anti-PD-1			0.12	0.003
25 μg ALG-031048				0.468

Table S5. Statistical analysis (P-value) of tumor weights in Hepa1-6 study presented in Figure S6B. The tumor weight on the last day of measurement (Day 36 after treatment start) was used to compare groups.

	P-Value			
	**Vehicle**	**10 mg/kg anti-PD-1**	**25 µg ALG-031048**	**100 µg ALG-031048**
Vehicle		0.002	<0.001	<0.001
10 mg/kg anti-PD-1			0.12	<0.001
25 µg ALG-031048				0.11

Table S6. Statistical analysis (P-value) of time to endpoint in CT26 bearing mice treated with ALG-031048 and/or anti-CTLA-4 presented in Figure 6. The time (days) between start of treatment and endpoint (TV ≥ 2000 mm^3) was used to compare groups. If the endpoint was not reached by the end of the study, the last day of measurement (Day 40) was used instead.

	P-Value			
	**Vehicle**	**25 µg ALG-031048**	**anti-CTLA-4**	**25 µg ALG-031048 + anti-CTLA-4**
Vehicle		<0.001	0.169	<0.001
25 µg ALG-031048			0.115	0.003
anti-CTLA-4				<0.001

Table S7. Statistical analysis (P-value) of time to endpoint in CT26 bearing mice treated with ALG-031048 administered SC or IT presented in Figure 7 A and B. The time (days) between start of treatment and endpoint (TV ≥ 2000 mm^3) was used to compare groups. If the endpoint was not reached by the end of the study, the last day of measurement (Day 38) was used instead.

	P-Value				
	**Vehicle**	**1 mg/kg ALG-031048 SC**	**25 µg ALG-031048 IT**	**4 mg/kg ALG-031048 SC**	**100 µg ALG-031048 IT**
Vehicle		0.002	<0.001	<0.001	<0.001
1 mg/kg ALG-031048 SC			0.214		0.003
25 µg ALG-031048 IT					
4 mg/kg ALG-031048 SC					0.476

Table S8. Statistical analysis (P-value) of tumor volume in MC38-hPD-L1 bearing mice treated with ALG-031048, atezolizumab or a combination thereof (Figure 7 C, D and E). The tumor volume on Day 11 was used to compare groups.

	P-Value			
	**Vehicle**	**5 mg/kg Atezolizumab**	**0.5 mg/kg ALG-031048**	**5 mg/kg Atezolizumab + 0.5 mg/kg ALG-031048**
Vehicle		<0.001	<0.001	<0.001
5 mg/kg Atezolizumab			0.123	0.017
0.5 mg/kg ALG-031048				<0.001

Table S9. Statistical analysis (unpaired t-test) of plasma cytokine levels in CT26 tumor-bearing mice after intratumoral (IT) or subcutaneous (SC) administration of ALG-031048 (Fig S8). 10 mice per group were treated with 3 doses 3 days apart. Plasma samples were collected 6 hours after the last dose.

IFN-γ	P-Value			
	Vehicle	25 µg ALG-031048 IT	1 mg/kg ALG-031048 SC	4 mg/kg ALG-031048 SC
Vehicle		0.0247	<0.0001	<0.0001
25 µg ALG-031048 IT			0.2317	0.007
1 mg/kg ALG-031048 SC				<0.0001

IFN-β1	P-Value			
	Vehicle	25 µg ALG-031048 IT	1 mg/kg ALG-031048 SC	4 mg/kg ALG-031048 SC
Vehicle		0.0087	<0.0001	<0.0001
25 µg ALG-031048 IT			0.035	<0.0001
1 mg/kg ALG-031048 SC				<0.0001

IL-6	P-Value			
	Vehicle	25 µg ALG-031048 IT	1 mg/kg ALG-031048 SC	4 mg/kg ALG-031048 SC
Vehicle		0.0087	<0.0001	<0.0001
25 µg ALG-031048 IT			0.035	<0.0001
1 mg/kg ALG-031048 SC				<0.0001

IL-12(p40)	P-Value			
	Vehicle	25 µg ALG-031048 IT	1 mg/kg ALG-031048 SC	4 mg/kg ALG-031048 SC
Vehicle		0.0962	0.9355	0.0962
25 µg ALG-031048 IT			0.0939	>0.9999
1 mg/kg ALG-031048 SC				0.0939

IP-10	P-Value			
	Vehicle	25 µg ALG-031048 IT	1 mg/kg ALG-031048 SC	4 mg/kg ALG-031048 SC
Vehicle		<0.0001	<0.0001	<0.0001
25 µg ALG-031048 IT			0.5759	0.8388
1 mg/kg ALG-031048 SC				0.447

MCP-1	P-Value			
	Vehicle	25 µg ALG-031048 IT	1 mg/kg ALG-031048 SC	4 mg/kg ALG-031048 SC
Vehicle		0.0024	0.0004	<0.0001
25 µg ALG-031048 IT			0.0051	<0.0001
1 mg/kg ALG-031048 SC				0.0037

MIP-1α	P-Value			
	Vehicle	25 µg ALG-031048 IT	1 mg/kg ALG-031048 SC	4 mg/kg ALG-031048 SC
Vehicle		<0.0001	<0.0001	<0.0001
25 µg ALG-031048 IT			0.0829	<0.0001
1 mg/kg ALG-031048 SC				0.0013

TNF-α	P-Value			
	Vehicle	25 µg ALG-031048 IT	1 mg/kg ALG-031048 SC	4 mg/kg ALG-031048 SC
Vehicle		0.0001	<0.0001	<0.0001
25 µg ALG-031048 IT			0.1837	0.0004
1 mg/kg ALG-031048 SC				0.0014
